# Employee Engagement and Innovative Work Behavior Among Chinese Millennials: Mediating and Moderating Role of Work-Life Balance and Psychological Empowerment

**DOI:** 10.3389/fpsyg.2022.942580

**Published:** 2022-07-15

**Authors:** Hazem Ali, Min Li, Xunmin Qiu

**Affiliations:** ^1^School of Economics and Management, Yiwu Industrial and Commercial College, Yiwu, China; ^2^School of Innovation and Entrepreneurship, Wenzhou University of Technology, Wenzhou, China

**Keywords:** employee engagement, innovative work behavior, work-life balance, psychological empowerment, Chinese millennials

## Abstract

Given the ever-changing business environment, organizations are forced to consider innovation as an essential prerequisite to enhance their efficiency, productivity, and sustainability. In this regard, organizations pay increased attention to enhancing employees' engagement (EE) and stimulating their innovative work behaviors (IWBs). Research emphasizes the importance of employees' IWBs in achieving competitive advantages and organizational sustainability. In this research, we address the question of whether employee engagement leads to stimulating IWBs of the Chinese millennial workforce in service industries. In addition, we explore the potential mediating effect of work-life balance (WLB) and the moderating influence of psychological empowerment (PE) on the relationship between EE and IWBs. Self-administered questionnaires were used to collect data from 372 Chinese senior employees working in the IT, trade, real estate, financial, and telecommunication industries. Our empirical findings showed that highly engaged employees are most likely to exhibit IWBs and maintain a WLB. In addition, the relationship between EE and IWB was partially mediated by WLB. Moreover, the interaction between EE and PE was found to strengthen employees' IWBs. Our study contributes to understanding the importance of EE as an essential prerequisite for millennials' IWBs and provides new insights for service organizations to encourage employees' IWBs. This study contributes to the human resource management field by offering valuable implications vis-à-vis how service organizations operating in a turbulent business environment stimulate the IWBs of their millennial workforce.

## Introduction

The continued negative impact of the COVID-19 pandemic has resulted in deteriorating employees' working conditions, increasing psychological anxiety, and the possibility of losing their jobs (Jin et al., 2022). In such business contexts, organizations need to adopt innovative processes and techniques to ensure adaptability and sustainability. Organizational innovation is a wide area covering R&D, marketing, processes, products, and managerial innovation. Recognizing the influential role of employees in service industries, we focus mainly on employees' IWBs. The importance of IWB is well-established in organization and human resources management literature. For instance, Li et al. (2019) underlined that is essential for building organizational sustainability and achieving competitive advantages while Anderson et al. (2014) argued that organizational success is dependent on understanding the factors enhancing employees' IWB.

Former research as undertaken by Scott and Bruce (1994) revealed that IWB is closely related to maintaining organizational strategic survival in social and economic environments and called for more research on determining the individual and organizational factors facilitating IWB. Similarly, De Jong and Den Hartog (2010) argued that IWB is essential not only for innovation-based companies or jobs but for all organizational members as well. Recognizing the importance of driving IWBs, researchers paid increasing attention to understanding its perquisites and predictors (Anderson et al., 2014; Tian et al., 2021).

In their attempt to stimulate and promote IWBs among their employees, managers face various challenges such as employees' readiness for innovation, discrepancies in the work environment, and the fact that differences in organizational cultures may hinder the generalization of the innovation-inducing behaviors (Tan et al., 2021). In this regard, we argue that EE has the potential to facilitate employees' IWBs. The concept of EE has received wide recognition from academicians and practitioners in the recent past (Saks and Gruman, 2014; Bakker and Albrecht, 2018; Sahni, 2021). The majority of research on work engagement and creativity was undertaken in a western context only (Hui et al., 2020). Mansoor et al. (2021) argued that organizational innovation and competitiveness depend mainly on the presence of an engaged and innovative workforce. Despite the vast research on employee engagement, there have been limited studies on this subject within the context of millennial employees (Sahni, 2021).

According to Bakker and Albrecht (2018) highly engaged employees to contribute significantly to increasing productivity and maintaining high levels of satisfaction, citizenship behavior, and performance. Schaufeli et al. (2002) defined work engagement as an employee's positive, fulling, and work-related state of mind characterized by dedication, vigor, and absorption. Extant literature lacks consensus on the antecedents and outcomes of employee engagement (Saks and Gruman, 2014; Bailey et al., 2017). In their meta-analysis of 130 studies, Borst et al. (2020) indicated that EE is positively related to two fundamental attitudinal outcomes: commitment and job satisfaction, and negatively associated with two behavioral outcomes: turnover intentions and Workaholism. Sahni (2021) found that EE was positively related to organizational commitment and negatively related to turnover intentions among millennials in Saudi Arabia. In their research on EE in UAE, Al Mehrzi and Singh (2016) called for more research on the probable antecedents and outcomes of EE. Our research extends the research on IWBs as a consequence of EE among the millennial workforce in the Chinese service industry.

Employees' daily schedule is divided between work and life, thus organizations need to consider the potential conflict between work time and time devoted to employees' life aspects such as family and other responsibilities or activities. Research indicated the importance of WLB in nurturing employees' wellbeing (Kinnunen et al., 2014). In this respect, many scholars advocated the importance of WLB for employees. Organizations pay increasing attention to improving employees' wellbeing and performance through appreciating the general canvases of employees' lives (Timms et al., 2015). Kinnunen et al. (2014) argued that the increase in women's participation in the workforce, changes in family structure, and technological changes have resulted in blurring the line between individual work life and personal life. In this regard, WLB is viewed as a major factor to stimulate favorable employee outcomes.

The concept of psychological empowerment has gained wide recognition among human resource management scholars due to its impact on enhancing favorable employee outcomes. Psychologically empowered individuals have the willingness and capability to act independently and to facilitate proactive behavior in meaningful ways (Thomas and Velthouse, 1990; Spreitzer, 1995). Spreitzer et al. (1999) argued that psychological empowerment has a positive impact on enhancing employees' feeling of self-efficacy, meaningfulness in their work, competence and active orientation toward their work. Bordin et al. (2006) underlined that PE can increase organizational commitment and job satisfaction among Singaporean IT employees. This research builds on the definition of PE presented by Spreitzer et al. (1999) as “intrinsic motivation manifested in four cognitions reflecting an individual's orientation to his or her work role: meaning, competence, self-determination, and impact”. In their definition of PE, Pieterse et al. (2010) described it as a psychological state residing within an individual and reflecting an active orientation toward his/her work role. This study understands PE as a motivational construct to stimulate and enhance the IWB of engaged employees. In this regard, PE covers four main areas namely employees' perceived abilities to perform their tasks effectively, selecting regulating actions, influencing the surrounding work environment, and undertaking meaningful job tasks.

In this research, we seek to address the question of whether EE facilitates IWB and WLB among millennials. In addition, we tend to explore the potential mediating role of WLB and the moderating role of PE in the relationship between EE and IWBs. Thus, this research contributes to research on the IWB as an outcome of EE by offering valuable implications regarding how service companies can stimulate the IWB of their employees in order to cope with and respond to the intensely dynamic business climate in the post-COVID-19 era. More specifically, our study highlights the importance of EE in facilitating WLB and driving employees' IWB. Furthermore, this study predicts that the relationship between EE and IWB is mediated by WLB and moderated by PE. Our empirical findings offer a profound academic reference for future research on EE and IWB. Despite the maturity of the employed constructs, to the best of our knowledge, this is the first study proposing and examining the hypothetical relationships among the study variables with emphasis on the millennial workforce's context.

## Theoretical Background and Hypothesis Development

### Innovative Work Behavior

Given the importance of IWB for organizational success, scholars pay increasing attention to understanding its antecedents and motivational factors to understand innovativeness at the individual level (Wu et al., 2014). According to Grossan and Apaydin (2010), the factors influencing IWBs are divided into organizational (or environmental) and individual factors. The current study employs WLB and PE as organizational factors, and EE as individual factor to understand IWBs among Chinese millennials in service industry.

De Jong and Den Hartog (2010) advocated the influential role of employees in exhibiting IWB through going beyond organizational routines, finding new ways to perform their job tasks, and relying on current technologies. Employees maintaining IWBs can appropriately and promptly interpret emerging work situations and provide new ideas to improve products and services (Afsar et al., 2018). Research on millennials' IWB in the Chinese context is limited and scattered. For instance, Hui et al. (2020) argued that Chinese millennials' IWB is positively driven by their organizational identification and work engagement plays a positive mediating role while Tian et al. (2021) found that employee creativity was positively related to prosocial motivation. Millennials have the advantage of accepting new ideas and exerting more effort to be creative and acquire new knowledge and skills when facing a challenge (Zhu et al., 2018). Accordingly, we argue that creative millennials maintain confidence and capabilities to offer innovative solutions to emerging work-related problems. Given their close association with the millennia era and digital development, millennials refer to individuals who were born between 1980 and 1994 (Levenson, 2010). In a flexible working environment, millennials maintain high self-confidence and prefer job autonomy to effectively accomplish their tasks autonomy (Kong et al., 2016).

This research builds on Spiegelaere, De Gyes, Van Witte, De Niesen, Hootegem and (2014) definition of IWB is the ability of employees to generate, introduce, and apply new and beneficial ideas, processes, procedures, or products. Such definition is applicable to the boundaries of a job role, group or department, or the whole organization. De Jong and Hartog (2007) argued that employees' IWB plays a fundamental role in enabling the organization to innovate and adapt to dynamic business environments through building and maintaining competitive advantages (Choi et al., 2016). Based on such argumentations, it is essential to examine the factors that develop and improve IWB from the employee perspective.

### Employee Engagement and Innovative Work Behavior

The research stream of EE has presented a set of favorable consequences such as job satisfaction, organizational citizenship behavior, organizational commitment, knowledge sharing (Bailey et al., 2017), and employee performance (Khusanova et al., 2021). Mansoor et al. (2020) argued that EE has a direct impact on IWBs and a significant mediating impact on the relationship between inclusive leadership and IWBs among Singaporean IT employees. Prior research indicated that EE has a significant positive influence on employees' IWB (Arifin et al., 2021). EE has a direct relationship with employee creativity among marketing personnel in the Pakistani context (Inam et al., 2021). Svensson et al. (2021) underlined that EE is positively related to IWB.

EE is a fundamental antecedent of IWB (Miller and Miller, 2020). Al-Ajlouni (2021) found that employees who are more engaged with their jobs are most likely to exhibit IWB. Similarly, Gemeda and Lee (2020) indicated that work engagement has a positive impact on task performance and IWB. Kahn (1990) explained employee engagement as the physical, cognitive, and emotional dedication of employees to perform their job tasks. In line with Kahn's conceptualization, researchers used different terms for EE such as work engagement, employee engagement, role engagement, and job engagement. Schaufeli and Bakker (2010) argued that work engagement and employee engagement are the most commonly used conceptualization of engagement in extant literature which are used interchangeably and stressed the need to distinguish between them. EE focuses on the link between an employee and his/her work and his/her organization while work engagement applies to assessing the relationship between an employee and his/her work only (Schaufeli and Bakker, 2010). This study intended to examine the impact of EE on IWB from the perspective of employees. Accordingly, we focused mainly on EE through examining the potential impact of employee's relationship with work and with their companies on their IWBs.

*Hypothesis 1*: Highly engaged employees are most likely to exhibit innovative work behaviors.

### Employee Engagement and Work-Life Balance

Researchers argued that EE has the potential to facilitate employees' WLB. For instance, Halbesleben et al. (2009) found that highly engaged employees have lower levels of work interference with family while Culbertson et al. (2012) showed that EE has a positive impact on family life through facilitating work-to family relationship. Karatepe and Demir (2014) indicated that employees with high levels of work engagement are able to integrate their work and family roles easily. Similar studies were undertaken by Marais et al. (2014) and Qing and Zhou (2017) revealed that work-family enrichment is significantly driven by EE.

Chen and Huang (2016) underlined that EE is closely related to predicting employees' IWBs. In the Chinese context, Qing and Zhou (2017) indicated that EE is an influential antecedent of work-family enrichment. In their research on engagement level among Chinese banking employees, Ilies et al. (2017) found that EE has an appositive impact on work–family interpersonal capitalization, which eventually leads to family satisfaction and achieving a balance between work and family.

In their review of the empirical research on the relationship between work engagement and WLB, Wood et al. (2020) argued that both constructs have a reciprocal association. This study builds on the research stream which views work engagement as an antecedent of WLB. We argue that engaged employees are more likely to maintain a balanced work-life. Our research extends Wood et al. (2020) research through the empirical examination of the impact of EE on WLB and IWBs. In addition, we examine the potential mediating impact of WLB on the relationship between EE and IWBs. This study intended to extend research on the outcomes of EE. More specifically, our study aims to examine the impact of EE on innovative workplace behavior and WLB among the millennial workforce in the Chinese context. Based on such argumentations, we predict that highly engaged employees are most likely to achieve a balance between their work and life and derive the following hypothesis.

*Hypothesis 2*: Employee engagement has a positive impact on WLB.

### Work-Life Balance and Innovative Work Behavior

Research presented numerous favorable employee outcomes of WLB such as improving job performance (Campo et al., 2021), enhancing employees' psychological wellbeing (Haider et al., 2018), and reducing turnover intention (Kerdpitak and Jermsittiparsert, 2020). Employees' IWB requires maintaining a highly focused state of mind and full engagement (Pieterse et al., 2010). Highly engaged employees are characterized by enthusiasm, continuous focus on work, and extra energy (Aryee et al., 2012) which enable them to take several initiatives, including innovative behavior (Eva et al., 2019). WLB is framed as an individual's satisfaction with multiple roles in work and personal life (Clarke et al., 2004). Kim and Yun (2019) found that employees' WLB has a positive impact on their IWBs in the Chinese hotel industry. Hence, we predict that employees maintaining a WLB are most likely to exhibit IWBs and posit the following hypothesis:

*Hypothesis 3*: Employee work-life balance has a positive impact on IWBs.

### Mediating Effect of Work–Life Balance

Academicians and practitioners advocate the important mediating role of WLB in organizational factors to improve organizational performance (Stankevičiene et al., 2021). Haar (2013) finds that the impact of family conflict and enrichment on employees' achievement and wellbeing outcomes was mediated by WLB. WLB has a positive link with organizational effectiveness, employees' attitudes, and behaviors, as well as employees' wellbeing (Au and Ahmed, 2014). Employees who lack balance between work and life due to family problems or excessive workload usually feel stressed at work and maintain negative work attitudes, which, in turn, results in burnout (Lawson et al., 2013). Nabawanuka and Ekmekcioglu (2021) argued that WLB has a significant effect on millennials' wellbeing and has a mediating impact on the relationship between perceived supervisor support and employee wellbeing. Rashmi and Kataria (2021) indicated that WLB was positively related to nursing professionals' job satisfaction and had a partial mediating role between the two job resources job autonomy and supervisor support; and job satisfaction.

The research presents a reciprocal relationship between EE and WLB because work and family life require a remarkable amount of energy, time, and emotional effort (Halbesleben, 2010; Timms et al., 2015). A high level of WLB in an organization enables workers to control their performance, such as allocating their working time efficiently. According to our knowledge, no studies have analyzed the potential impact of WLB on the relationship between EE and IWB. Based on the above-mentioned research, WLB has the potential to mediate the relationship between EE and IWB. Hence, we derive the following research hypothesis:

*Hypothesis 4*: WLB has a mediating effect on the relationship between EE and IWB.

### Moderating Role of Psychological Empowerment

Researchers have employed PE as a moderating variable when examining innovative behavior in relation to different leadership styles. For instance, Pieterse et al. (2010) underlined that high PE strengthens the positive impact of transformational leadership on the innovative behavior of government employees in the Netherlands. Similarly, Grošelj et al. (2020) showed that PE moderates the relationship between leadership (authentic as well as transformational leadership) and IWB. Companies need to consider employees' willingness to exhibit IWBs work behaviors as their formal job requirements do not motivate them to exhibit such extra role behavior (Huhtala and Parzefall, 2007). We argue that PE has the potential to stimulate IWBs of engaged millennial employees.

Researchers argued that Asian employees exhibit limited IWBs due to challenges made by their leaders and limited collective support (Zuraik and Kelly, 2019). Accordingly, we decided to use PE as a moderating variable, instead of mediating variable, to examine whether the presence of PE strengthens the relationship between EE and IWB. Employees perceiving high PE are prepared to adjust their behaviors and actively get engaged in exhibiting IWBs (Afsar et al., 2014). Psychologically empowered employees value their work and maintain intrinsic motivation, which stimulates their IWB (Krishnan, 2012). Leaders need to energize the dimensions of PE and instill employees' free will in translating organizational vision and mission into work context and daily routine tasks (Bin Saeed et al., 2019). In another study, Khan et al. (2021b) indicated that servant leadership has a positive impact on PE, job crafting, and employees' IWB and argued that PE has a positive mediating influence on the relationship between servant leadership and IWB. García-juan and Escrig-tena (2018) showed that employee empowerment has a significant impact on his/her responsiveness to customer needs and dealing cordially with customers which ultimately results in elevating firm performance. We argue that EE is more effective in driving IWB when PE is high. On the contrary, engaged employees with limited PE, lack the confidence to take innovative initiatives which may result in discouraging IWB. Therefore, we predict that PE has a significant moderating influence on the relationship between EE and IWB and posit the following hypothesis:

*H5: Psychological Empowerment Significantly Strengthens the Influence of employee engagement on Innovative Work Behavior*.

**Figure 1:**
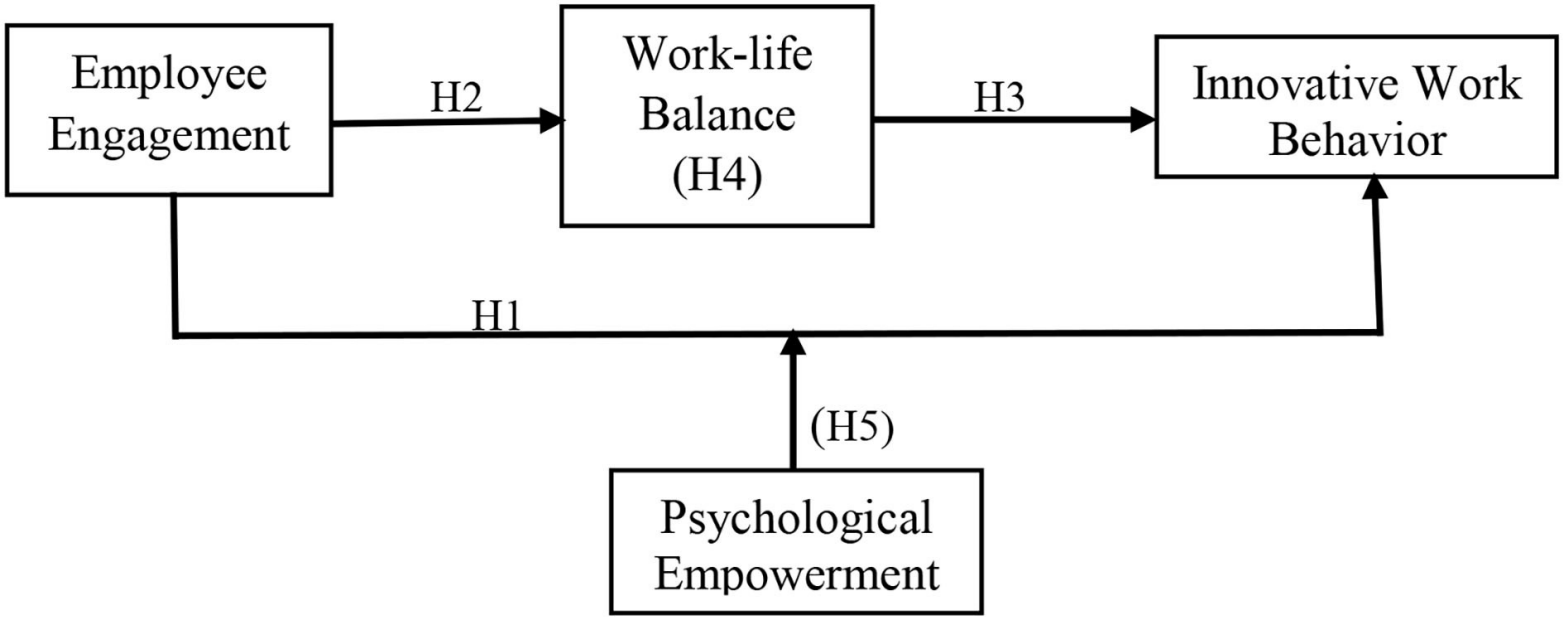
Research model.

Figure 1 depicts the proposed research model. As previously mentioned, the current study predicts a direct positive impact of EE on employees' IWB and WLB. In addition, the potential relationship between EE and IWB is expected to be mediated by WLB and moderated by PE.

## Methods

### Sample and Data Collection

Our study's targeted populations were senior employees working in Chinese IT, trade, real-estate, financial, and telecommunication companies located in four main cities in Zhejiang province: Hangzhou, Ninbo, Jinhua, and Wenzhou. Such industries are frequently employed as the research context for service industry research (Zhou et al., 2018; Khan et al., 2021b; Sahni, 2021; Tian et al., 2021). In addition, employees working in such service industries need to possess the skills and capabilities to exhibit IWB through interaction with customers.

Recognizing their proactive personalities, millennials express more innovative behavior than the previous generation (Giebels et al., 2016), especially in knowledge-based economies (Hui et al., 2020). Millennials accept new ideas, exert exceptional efforts to acquire skills and knowledge, and tend to take risks to achieve short-term benefits (Zhu et al., 2018). The Chinese workforce is dominated by millennials with the potential of playing a fundamental role in achieving organizational innovation and competitiveness (Zhao, 2018; Asif et al., 2019; Hui et al., 2020). Chinese millennial workforce seeks higher pay and flexibility when compared to generations X and Y (Lin et al., 2015). Accordingly, leaders need to train and empower their millennial workforce to exhibit IWBs to maintain a competitive position, especially in the context of service industries.

Upon the identification of major and big-sized branches of target service companies, random sampling was employed to select the study sample. Following Tian et al. (2021) data collection procedure, we contacted branch managers through the organizations' service center and explained and requested a face-to-face meeting or personal contact detail of branch managers. Then, we explained the purpose of our study and requested managers' consent to assist us in the data collection process through telephonic or face-to-face interviews. After a lengthy communication with managers, we managed to convince 39 branch managers out of a total of 46 branches to provide assistance in data collection. We identified three sampling criteria and explain them carefully to branch managers: employees should be aged born between 1981 and 1996, employees hold a senior position and capabilities to show innovative behaviors in fulfilling their job tasks. The overall distribution and collection of questionnaires were conducted from November 2021 to March 2022.

In order to stimulate respondents' consent to fill in the questionnaires, we used printed and self-administered questionnaires. We hired two local citizens to ensure a smooth and effective data collection process. In this regard, we explained the purpose of our study, identified the names and branches of targeted companies, and provided sufficient training on how to administer and collect the questionnaires. Symbolic gifts were given to managers to motivate their employees to fill in a survey questionnaire and return it in a reasonable time. Respondents were given 2 weeks to fill in the questionnaires. We used WeChat or in-person visits to communicate and follow up with respondents. A total of 600 questionnaires were distributed with only 372 being complete and valid for analysis, representing a 53% response rate. Such a moderate response rate might be justified based on the required sampling criteria. However, extant literature shows the acceptance and utilization of low response rates such as 41% (Sahni, 2021), 34% (Culbertson et al., 2012), and 31% (Gemeda and Lee, 2020).

### Measures

This study used mature and well-established measurement scales. Appendix 1 presents a detailed description of the employed measurement scales in the survey. A five-point Likert scale ranging from 1 “strongly disagree” to 5 “strongly agree” was used to assess the adopted measures and Cronbach alpha values were calculated to gauge the constructs' reliability. The independent variable, employee engagement was measured using nine items developed by Schaufeli et al. (2006) with a reported reliability value (*a* = 885). As a multidimensional construct, IWB involves employees' behaviors contributing to enhancing innovative processes (De Jong and Hartog, 2007; Saeed et al., 2019). Innovative work behavior was measured using Ma Prieto and Pérez-Santana (2014) scale. This measurement scale has four items with a reported high-reliability value (*a* = 0.810) and (*a* = 0.93) by Mishra et al. (2019).

The moderating variable, psychological empowerment was assessed using Spreitzer's (1995) measurement scale which includes 12 items including four dimensions with a Cronbach Alpha Coefficient (0.843). The meaning dimension refers to the perceived value of employees' roles in the workplace based on his/her standards. The competence dimension defines an employee's belief in possessing essential capabilities to perform a set of tasks. Effect dimensions determine the perceived degree to which an employee can affect the operative, strategic, and administrative work outcome. Finally, the self-determination dimension refers to a perceived sense of freedom and job autonomy. The mediating variable, work-life balance was measured using four items developed by Hayman (2005). Control variables were employed to gain a detailed explanation of research findings such as the employee's gender, age, educational level, and organizational tenure. This research build's on Dimock's (2019) definition of millennials as “anyone born between 1981 and 1996”.

## Results

Table 1 summarizes the descriptive information of the research participants.

**Table 1:** Descriptive information (*N* = 372).

	**Frequency**	**Percent**
Gender	Male	227	61
	Female	145	39
Age	<30	55	14.8
	30–35	112	30.1
	36–40	119	32
	More than 40 years	86	23.1
Industry	IT	66	17.8
	Trade	49	13.2
	Real-estate	83	22.3
	Financial	79	21.2
	Telecommunication	95	25.5
Education	Diploma	27	7.2
	Graduate	264	71
	Post-Graduate	82	22
Organizational tenure (years)	0–2	26	7
	2–4	96	26
	4–6	133	36
	6–8	60	16
	8–10	41	11
	More than 10 years	16	4

Out of 372 valid samples, 61% were male, and the remaining 39% were female. For educational qualifications, 71% hold undergraduate degrees followed by 22% with postgraduate degrees. Regarding employees' ages, approximately 32% aged between 36 and 40 years, and 31% aged between 30 and 35 years. The unit of analysis for this research included millennial employees working in different service industries. Findings showed that approximately 26% of the valid sample were working in telecommunication, 22% in real estate and 21% in the financial industry. The majority of participants have organizational tenure ranging between 6 and 8 years (36%) and 4 and 6 years (26%).

### Model Analysis

Data analysis was conducted using PLS-SEM due to its wide usage in business and management fields and providing a fully developed and comprehensive system of variance (Matthews et al., 2018). A two-step approach was used for PLS-SEM. The first step presented the evaluation of the structural model by assessing reliability, validity, and common method bias. The second step introduced the results of the structural models using cross-validated redundancy, path coefficients, and coefficient of determination.

### Assessment of the Measurement Model

This study employed a reflective-formative method for PE construct. Accordingly, the proposed model was assessed using first-order and second-order. Factor loadings, internal consistency, and validity results were used to assess the measurement model. Table 2 summarizes the constructs, correspondent item codes, and descriptive statistics. All the factor loadings of constructs' items were higher than 0.7 and the reported Cronbach's alpha and composite reliability were greater than 0.7 (Hair et al., 2017). Accordingly, the reliability of constructs was retained. Convergent and discriminant validity were assessed based on the reported average variance extraction (AVE) and inter-correlations among constructs (Fornell and Larcker, 1981). Findings showed that the AVE of study constructs was higher than 0.5 (Table 2). Further, the AVE's square root for each construct was higher than the inter-correlations of the variables with other model variables.

**Table 2:** Measurement model.

**Construct**	**Item code**	**Loading**	**CA**	**CR**	**AVE**	**Inner VIF**
EE	EE1	0.831	0.928	0.940	0.636	1.694
	EE2	0.738				
	EE3	0.788				
	EE4	0.773				
	EE5	0.802				
	EE6	0.800				
	EE7	0.838				
	EE8	0.797				
	EE9	0.808				
WLB	WLB1	0.815	0.810	0.875	0.637	1.859
	WLB2	0.857				
	WLB3	0.755				
	WLB4	0.760				
PE	PE1	0.889	0.953	0.959	0.660	2.714
	PE2	0.932				
	PE3	0.905				
	PE4	0.869				
	PE5	0.836				
	PE6	0.858				
	PE7	0.949				
	PE8	0.944				
	PE9	0.928				
	PE10	0.936				
	PE11	0.942				
	PE12	0.911				
IWB	IWB1	0.841	0.828	0.886	0.661	2.823
	IWB2	0.810				
	IWB3	0.732				
	IWB4	0.861				

To ensure the non-existence of CMB, we employed Harman's single factor test and full collinearity method. The results of Harman's singly factor test indicated that a single factor explained only 39.49% of the total variance which is below the 50.0% threshold (Podsakoff et al., 2003). The full collinearity method used the variance inflation factor (VIF) to ensure that data is free from CMB. Table 2 indicates that the reported VIFs values were lower than 3 as recommended by Hair et al. (2011). Further, the possibility of CMB was assessed using the correlation-matrix procedure (Table 3). Correlation results showed that the correlation among constructs is less than 0.9 (Tehseen et al., 2020). Accordingly, our data is free from CMB.

**Table 3:** Latent variable correlation and square root of AVE.

	**Gender**	**Age**	**Tenure**	**EE**	**WLB**	**PE**	**IWB**
Gender							
Age	−0.139						
Tenure	−0.085	−0.026					
EE	−0.134	−0.031	−0.026	**0.797**			
WLB	−0.083	0.105	0.069	0.453	**0.807**		
PE	0.018	0.043	0.038	0.572	0.572	**0.798**	
IWB	−0.174	0.106	−0.019	0.211	0.179	0.475	**0.813**

*N = 372. EE, Employee Engagement; WLB, Work-Life Balance; PE, Psychological Empowerment; IWB, Innovative Work Behavior. Values on the diagonal (bold) are square root of the AVE while the off-diagonals are correlations*.

### Results of the Structural Model

**Figure 2:**
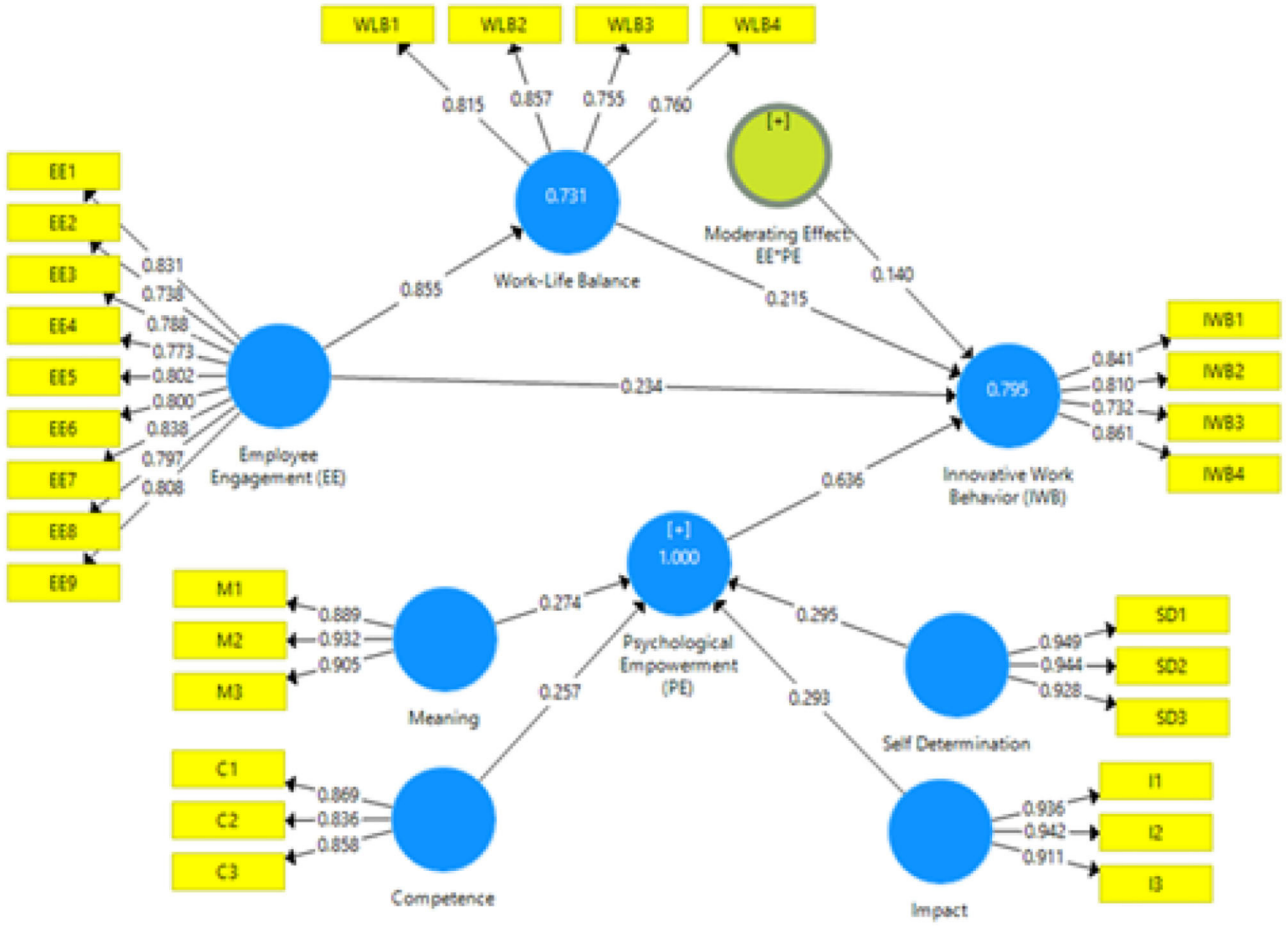
Results of structural equation modeling.

The proposed structural model was evaluated using coefficients of determination, path coefficients, *t*-value, and *p*-value. This research employed a bootstrapping method with 5,000 bootstraps as recommended by Hair et al. (2017) and 372 valid cases to generate path values and their significance level as proposed by Hensler et al. (2009). Figure 2 presents the assessment of the structural model. The reported *R*^2^-value of 0.731 indicates that 73.1% of the variation in WLB is explained by EE, while 79.5% of the variation of IWB is explained by EE, WLB, and PE. Coefficients of determination values of 0.60, 0.33, and 0.19 are considered substantial, moderate, and low (Cohen, 1998). It is evident that *R*^2^-values are substantial and reveal that independent variables have a significant impact on the dependent variable. Further, we employed effect sizes (f^2^) and (q^2^) to validate the results.

According to Cohen (1998), effect sizes values indicate the predictive relevance of an exogenous variable for a specific endogenous variable and can be categorized as high (0.35), medium (0.15), and low (0.02). The structural model was further reinforced by calculating the q^2^ to validate the predictive relevance of EE for WLB, and the relevance of EE, WLB, and PE for IWB through mediation and moderation analysis.

Hensler et al. (2009) proposed that the structural model has a significant predictive relevance when q^2^ > 0. The results of the study showed that the suggested model has medium and considerable predictive relevance as illustrated in Table 4. Further, the moderating findings of PE had a small effect size (with 0.022 effect size value; Table 5). Standardized root mean square residual (SRMR) is widely employed as an absolute measure of model fit. A model with a zero SRMR value is considered a perfect fit model while a model with less than 0.08 is considered as a good fit model (Hu and Bentler, 1999). Findings in Table 4 showed that our model maintains adequate goodness of fit.

**Table 4:** Model strength.

	**Effect size**			**Coefficient of determination**	
**Construct**	**SSO**	**SSE**	**Q^**2**^ (= 1–SSE/SSO)**	**R^**2**^**	**Adjusted R^**2**^**
WLB	372.00	238.371	0.362	0.731	0.728
IWB	372.00	271.662	0.269	0.795	0.786
	**f** ^**2**^	
	**WLB**	**IWB**	**IWB**
EE	0.593	0.133	0.034 (Small)
WLB		0.028	0.109 (Small)
EE → WLB → IWB	0.129		0.095 (Small)
EE* PE → IWB	0.097		0.086 (Small)

*N = 372. Goodness of fit: SRMR = 0.078; EE → PE. Moderation effect size is small. EE, Employee Engagement; WLB, Work-Life Balance; PE, Psychological Empowerment; IWB, Innovative Work Behavior*.

**Table 5:** Path coefficient and hypotheses testing.

**Hypothesis**		**Relationship**	**Path coefficient**	**SD**	**t-value**	***p*-value**	**Decision**
**Direct effect**	**H1**	**EE → IWB**	**0.234**	**0.058**	**2.136**	**0.040**	**Supported**
	H2	EE → WLB	0.855	0.063	26.292	0.000	Supported
	H3	WLB → IWB	0.215	0.049	2.157	0.029	Supported
Indirect effect	Mediating (H4)	WE → WLB → IWB	0.184	0.056	4.316	0.001	Supported
	Moderation (H5)	WE*PE → IWB	0.140	0.071	3.284	0.002	Supported

N = 372. EE, Employee Engagement; WLB, Work-Life Balance; PE, Psychological Empowerment; IWB, Innovative Work Behavior. *t > 1.96 (p < 0.05).

### Mediation Analysis

This study used intended to identify the mediating role of WLB on the relationship between EE and IWB. According to MacKinnon et al. (2002), the mediator variable has a significant mediating effect when the direct path between the independent variable and mediating variable is significant and the direct path between mediating variable and the dependent variable is significant. Findings showed a positive and significant direct path from EE to WLB (β = 0.609, *p* = 0.001) and from WLB to IWB (β = 0.382, *p* = 0.001). Guided by Matthews et al. (2018), the findings revealed that WLB plays a partial mediating role between EE and IWB (Table 5).

### Moderation Analysis

**Figure 3:**
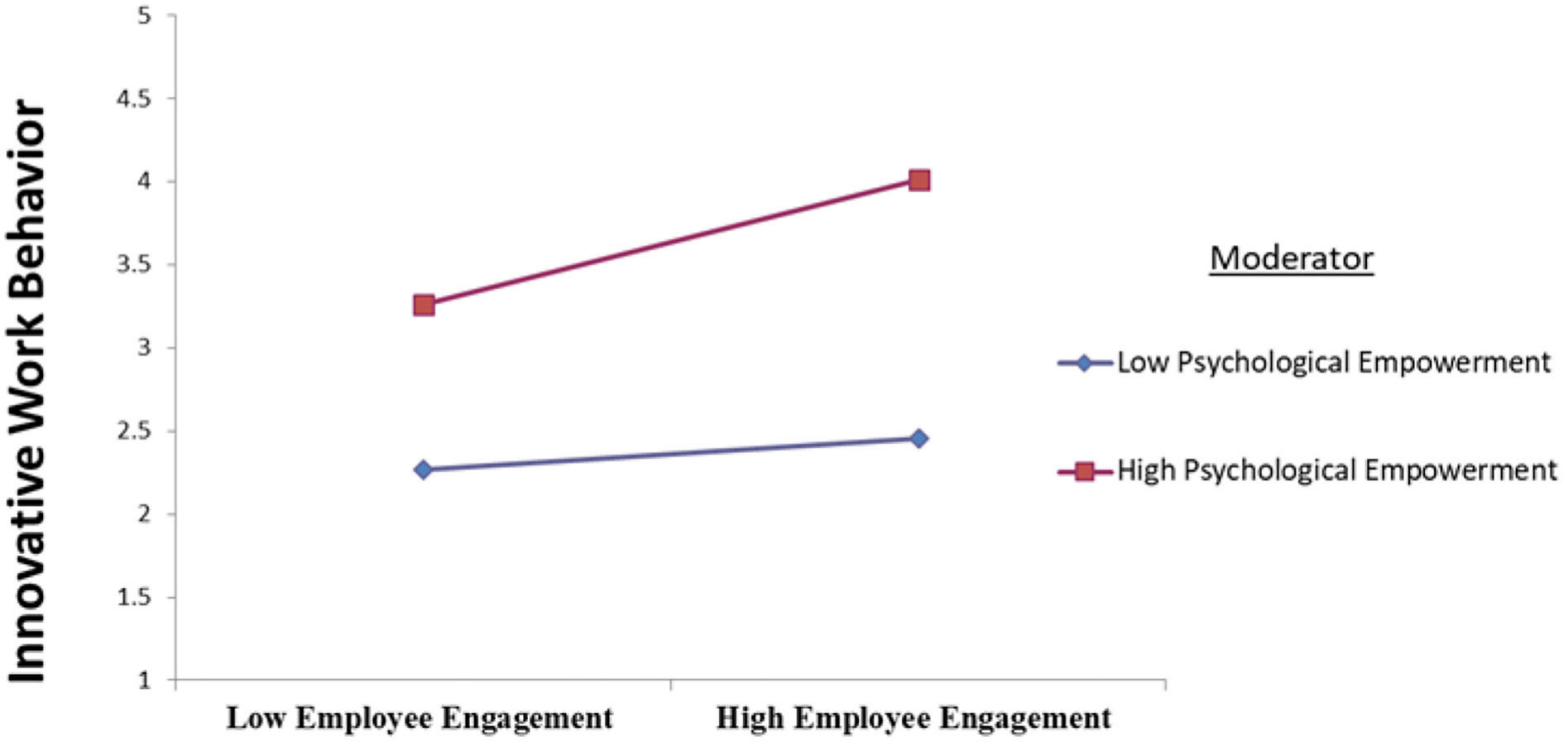
Visualization of moderation effect.

Given the continuous-based nature of the moderating variable, this research employed a product-indicator-method (PIM) using PLS-SEM to assess the moderating role of PE (Chin, 2010). Table 5 shows the results of moderating impact of PE. It is evident that the impact of interaction terms of EE and PE on IWB was significant (β = 0.140, *p* = 0.002). Accordingly, the hypothesis was accepted. The slope for the relationship between EE and IWB moderated by PE found that higher PE strengthens the relationship between EE and IWB (Figure 3). In other words, when PE is high, the influence of EE on IWB tends to be stronger.

## Discussion and Conclusion

Research findings showed that, as predicted, employee engagement was found to positively influence IWB among the millennial employees working in the service industry. Such a finding is consistent with extant literature (Mansoor et al., 2020; Arifin et al., 2021; Inam et al., 2021; Svensson et al., 2021). Similarly, Al-Ajlouni (2021) and Gemeda and Lee (2020) reported similar findings and advocated the relevance of EE in encouraging employees' IWBs.

In addition, the findings of our study have empirically validated the direction of causality between EE and WLB and concluded that EE is a fundamental antecedent of WLB. The majority of existing literature showed a positive impact of EE on WLB. For instance, Halbesleben et al. (2009) and Culbertson et al. (2012) found that employees with high work engagement are capable of maintaining a WLB. Similar views were shared by Karatepe and Demir (2014), Marais et al. (2014), and Qing and Zhou (2017) who argued that highly engaged employees are capable of integrating their work and family roles. However, some studies indicated that EE may lead to work-family conflict. For example, Chen and Huang (2016) stated that EE is positively related to burnout and high work-family conflict. Bakker et al. (2014) showed that EE has a positive influence on work–family facilitation, which results in enhancing employee's/family satisfaction.

Our empirical findings showed that WLB has a direct impact on IWBs. This finding is compatible with various studies (Pieterse et al., 2010; Aryee et al., 2012; Eva et al., 2019; Kim and Yun, 2019). Further, WLB was found to play a partial mediating role between EE and IWB. This indicated that highly engaged employees' IWBs increase when they achieve WLB. Existing literature employs WLB as a mediating variable to examine different employee attitudes and outcomes (Lawson et al., 2013; Nabawanuka and Ekmekcioglu, 2021; Rashmi and Kataria, 2021). To the best of our knowledge, our study presents an initial attempt to empirically validate the mediating role of WLB on the relationship between EE and IWB. WLB partially mediated the relationships between EE and IWBs. This suggests that WLB has a direct impact on employees' IWBs and an indirect influence on IWBs by affecting their level of work engagement. This implies that engaged employees who maintain PE in their work are most likely to exhibit IWBs.

Moreover, engaged employees who perceive PE exhibit IWBs. PE was found to strengthen the positive relationship between EE and IWB. Such finding is compatible with (Thomas and Velthouse, 1990; Pieterse et al., 2010; Grošelj et al., 2020) who argued that PE is important for IWB. Afsar et al. (2014) argued that PE is directly related to IWB.

### Managerial Implications

Our findings show that EE plays a fundamental role in enhancing IWB. This implies that engaged employees are more likely to induce IWBs. Accordingly, employers need to encourage EE by formulating relevant policies and programs in order to stimulate IWBs. Leaders need to understand the influential role of EE in driving employees' IWBs. Hence, identifying and implementing relevant and effective EE practices encourage employees to exhibit IWBs. Guided by our research findings, leaders in service industries are advised to ensure the effective engagement of millennial employees. This can be achieved through servant leadership. According to Khan et al. (2021a), servant leadership was found to positively influence the meaning and work engagement and ascertained the mediating role of meaning as a fundamental dimension of psychological empowerment in the relationship between servant leadership and work engagement among Pakistan employees working in the service sector.

Based on our findings, leaders need to emphasize EE practices in order to facilitate employees' WLB. Leaders need to pay increasing attention to creating a suitable work environment where employees maintain a work–life balance. This can be achieved by allowing employees to have flexible work hours and balancing family and work duties. Anderson et al. (2014) called for more research on integrating the innovation research, which, in turn, contributes significantly to the field of innovativeness. Given the reciprocal association between work engagement and WLB factors, as revealed by Babic et al. (2017) and Timms et al. (2015), more longitudinal investigations should be attempted in order to more clearly identify their associations.

Moreover, service organizations need to pay attention to developing and increasing employees' PE as an essential prerequisite for IWB. In this regard, we propose that EE practices should be integrated with PE practices to motivate and boost employees' IWBs. The moderating role of PE offers practical solutions on how to improve the IWBs of employees at an individual level. Managers can stimulate and promote IWB through the implementation of relevant practices to build and improve PE. In other words, leaders need to fully understand the antecedents of IWBs and consider encouraging EE and securing employees' PE as building blocks for stimulating employees' IWBs.

Prior research found that PE has a positive significant impact on employees' IWB (Thomas and Velthouse, 1990; Pieterse et al., 2010). Employees with high levels of PE perceive their job tasks as profoundly impactful and meaningful. Millennials respect their managers greatly and value working in an interpersonal harmonic atmosphere, thus they can get the required managerial support and gain access to resources to work hard (Lin et al., 2015). Managers' role in securing an interpersonal harmony allows millennials to focus on their work and trigger the potentiality of their creativity (Dong et al., 2016), which eventually leads to building and enhancing their creativity and enables them to gain the required support. This study establishes a boundary condition in terms of PE to the EE in stimulating IWB among employees in the service industry. Despite the wide research on the direct impact of PE on IWB, our study argues that PE is a precondition for IWB.

This research enriches our understanding of the importance of EE in driving innovative behaviors and facilitating WLB of the millennial workforce. Our empirical findings showed that EE implicates employees' WLB and IWBs. In view of the research findings, we propose the integration of PE practices which could encourage engaged employees to exhibit IWB related to solving existing or emerging problems or challenges in service companies. Nevertheless, managers should understand that the relationship between EE and IWB is mediated by WLB. We propose that leaders need to nurture the PE for engaged employees in order to stimulate their IWB. Zhao (2018) argued that millennials have transformed China's workforce and brought many challenges to human resource managers and stressed the need for companies to develop and implement structural and psychological empowerment for millennials. In today's innovativeness business world, leaders need to be aware of employees' intrinsic motivation, believe in their abilities, and allow them to decide freely on how to implement their work tasks.

### Limitations

The target population for this study involved millennial employees who were born around 1981–1996. The generalization of study findings might be challenged as the current study focused mainly on millennials working in the service industry in the Chinese context. This research relied mainly on data collected from employees' perspectives only. Hence, future studies may integrate employees' and managers perspectives to examine the impact of EE on IWB. In addition, further research may employ a larger sample size in different contexts to generalize the research findings. Recognizing China's cultural values and diverse perspectives such as high power distance and collectivism (Chen et al., 2020), the generalization of our empirical findings might be challenged. Therefore, future research may examine the applicability and extend the scope of our model in different contexts with larger samples and employ qualitative studies. Despite the above-mentioned research limitations, the empirical findings of our study contribute to the growing body of research on EE and IWBs.

## Data Availability Statement

The raw data supporting the conclusions of this article will be made available by the authors, without undue reservation.

## Ethics Statement

Ethical review and approval was not required for the study on human participants in accordance with the local legislation and institutional requirements. The patients/participants provided their written informed consent to participate in this study.

## Author Contributions

HA has take the responsibility of conceptualization, writing the original draft, project administration, reviewing, and editing the manuscript. ML has taken responsibility of data curation, formal, methodology, and resources. XQ has contributed to the design of this study, participated in writing the initial draft, take a shared responsibility for data curation and resources, and was accountable for his tasks and acknowledged his agreement with the final version of the manuscript. All authors have read and agreed to the published version of the manuscript.

## Conflict of Interest

The authors declare that the research was conducted in the absence of any commercial or financial relationships that could be construed as a potential conflict of interest.

## Publisher's Note

All claims expressed in this article are solely those of the authors and do not necessarily represent those of their affiliated organizations, or those of the publisher, the editors and the reviewers. Any product that may be evaluated in this article, or claim that may be made by its manufacturer, is not guaranteed or endorsed by the publisher.

## Appendix 1: Survey Questionnaire

**Table:**

**Construct**	**Item Code**	**Correspondent statement**	
Employee engagement (Schaufeli et al., 2006)	EE1	I feel bursting with energy at work.
	EE2	I feel strong and vigorous at work.	
	EE3	When I get up in the morning, I want to go to work.	
	EE4	I am enthusiastic about my work.	
	EE5	My work inspires me.	
	EE6	I am proud of my work.	
	EE7	When I work hard, I feel happy.	
	EE8	I am immersed in my work.	
	EE9	I get carried away when I am working.	
Work-life balance (Hayman, 2005)	WLB1	There is enough time for recreation activities	
	WLB2	I do not need to work overtime as I use to finish work within working hours	
	WLB3	I have enough time for my family and friends	
	WLB4	I value the social benefits that the company offers me	
Psychological empowerment (Spreitzer, 1995)	Meaning	PE1	The work I do is very important to me
		PE2	My job activities are personally meaningful to me
		PE3	The work I do is meaningful to me
	Competence	PE4	I am confident about my ability to do my job
		PE5	I am self-assured about my capabilities to perform my work activities
		PE6	I have mastered the skills necessary for my job
	Impact	PE7	My impact on what happens in my department is large
		PE8	I have a great deal of control over what happens in my department
		PE9	I have significant influences over what happens in my department
	Self Determination	PE10	I can decide on my own how to go about doing my work
		PE11	I have considerable opportunity for independence and freedom in how I do my job
		PE12	I can decide for myself how to organize my work
Innovative work behavior (Ma Prieto and Pérez-Santana, 2014)	IWB1	I show innovative and creative behaviors	
	IWB2	I am able to take the risk of being innovative and creative	
	IWB3	I am able to search for new working methods, techniques or instruments	
	IWB4	I am able to anticipate problems and opportunities	
